# Use of Mustard Extracts Fermented by Lactic Acid Bacteria to Mitigate the Production of Fumonisin B_1_ and B_2_ by *Fusarium verticillioides* in Corn Ears

**DOI:** 10.3390/toxins14020080

**Published:** 2022-01-21

**Authors:** Raquel Torrijos, Tiago de Melo Nazareth, Pilar Vila-Donat, Jordi Mañes, Giuseppe Meca

**Affiliations:** Laboratory of Food Chemistry and Toxicology, Faculty of Pharmacy, University of Valencia, Ave. Vicent Andrés Estellés s/n, 46100 Burjassot, Spain; raquel.torrijos@uv.es (R.T.); pilar.vila@uv.es (P.V.-D.); jorge.manes@uv.es (J.M.); giuseppe.meca@uv.es (G.M.)

**Keywords:** yellow mustard, oriental mustard, *Lactiplantibacillus plantarum*, biopreservation, mycotoxin, antifungal activity, *Brassica juncea*, *Sinapis alba*, fungi, antimycotoxigenic

## Abstract

Corn (*Zea mays*) is a worldwide crop subjected to infection by toxigenic fungi such as *Fusarium verticillioides* during the pre-harvest stage. *Fusarium* contamination can lead to the synthesis of highly toxic mycotoxins, such as Fumonisin B_1_ (FB_1_) and Fumonisin B_2_ (FB_2_), which compromises human and animal health. The work aimed to study the antifungal properties of fermented yellow and oriental mustard extracts using nine lactic acid bacteria (LAB) in vitro. Moreover, a chemical characterization of the main phenolic compounds and organic acids were carried out in the extracts. The results highlighted that the yellow mustard, fermented by *Lactiplantibacillus plantarum* strains, avoided the growth of *Fusarium* spp. in vitro, showing Minimum Inhibitory Concentration (MIC) and Minimum Fungicidal Concentration (MFC) values, ranging from 7.8 to 15.6 g/L and 15.6 to 31.3 g/L, respectively. Then, the lyophilized yellow mustard fermented extract by *L. plantarum* TR71 was applied through spray-on corn ears contaminated with *F. verticillioides* to study the antimycotoxigenic activity. After 14 days of incubation, the control contained 14.71 mg/kg of FB_1_, while the treatment reduced the content to 1.09 mg/kg (92.6% reduction). Moreover, no FB_2_ was observed in the treated samples. The chemical characterization showed that lactic acid, 3-phenyllactic acid, and benzoic acid were the antifungal metabolites quantified in higher concentrations in the yellow mustard fermented extract with *L. plantarum* TR71. The results obtained confirmed the potential application of fermented mustard extracts as a solution to reduce the incidence of mycotoxins in corn ears.

## 1. Introduction

The contamination of food and feedstuffs by mycotoxins currently remains a significant concern in developed countries, and it is estimated that between 5 and 10% of the world’s food supply is squandered because of fungal growth [1]. Moreover, depending on the mycotoxin of concern and the analytical method employed, the prevalence of mycotoxins in food grains might be 60–80% [2]. Thus, toxigenic fungi are, perchance, the most significant pathogens worldwide in terms of food safety [3,4].

Corn (*Zea mays*) is subjected to infection by fungi such as *Fusarium verticillioides* and *Aspergillus flavus* throughout the supply chain [5]. The contact of corn kernels with such toxigenic agents not only leads to grain quality diminishment and economic losses but also menaces the health of animals and consumers who are subject to mycotoxin ingestion through corn or derived foods [6]. *Fusarium* species are prevalent in the field and frequently invade and synthesize mycotoxins in a crop. Moreover, the inadequate pre-harvest procedures of small-holder farmers, along with favourable meteorological conditions, contribute to fungal growth and mycotoxin contamination after harvest [7,8]. Fumonisin B_1_ (FB_1_) is the major mycotoxin generated by *F. verticillioides*, being considered probably carcinogenic to humans, according to IARC, and along with fumonisin B_2_ (FB_2_), has become significant contaminants in the food and feed industries [9]. These mycotoxins are frequently found as single and co-contaminants in cereals or cereal-based food and feed products [10,11]. In addition, the synergistic or additive toxic effects of mycotoxins, established by multiple occurrences or co-occurrences, have been highlighted by several authors [12,13].

Synthetic antifungals have represented the most prevalent method of combating fungal spoilage due to their broad-spectrum action. However, their use presents several disadvantages [14]. Agrochemicals are associated with environmental challenges, due to their stability and toxicity, since they can accumulate over time. They are hazardous to aquatic creatures, and their concentration in stream water has grown significantly in recent years [15]. Fungicides are also related to carcinogenic, teratogenic, and irritant effects in various human organs [16,17], prompting researchers to develop novel techniques of food spoilage management that assure food safety without compromising human health. Among these, biopesticides (natural pesticide compounds) are considered a promising and sustainable solution because they can remove target pests and lead to minimal environmental pollution [18].

Yellow mustard (*Sinapis alba*) and oriental mustard (*Brassica juncea*) have been previously used as culinary seasonings. However, recently the antifungal properties of powdered mustard have been demonstrated in food [19,20]. Both species contain a high concentration of glucosinolates, which are cleaved by myrosinase (EC 3.2.1.147) in the presence of moisture and an acidic pH, producing isothiocyanates as well as thiocyanates, nitriles, and a few other minor chemicals. The myrosinase synthesizes *p*-hydroxybenzyl isothiocyanate (*p*-HBIT) from sinalbin, the predominant glucosinolate in yellow mustard. In contrast, allyl isothiocyanate (AITC) is synthesized from sinigrin, the main glucosinolate in oriental mustard [21].

Biopreservation is a natural process that uses microbes, or their antimicrobial active metabolites, to prolong the shelf life and increase the safety of foods. Recently, authors have suggested the use of lactic acid bacteria (LAB) as an alternative to synthetic biocides for preventing fungal growth [22,23]. Additionally, it is critical to mention that the majority of LAB are widely acknowledged as safe and have QPS (qualified presumption of safety), so they can be considered excellent candidates for their use as natural preservatives in food and feedstuff [24]. However, no reports demonstrated the antifungal capacity of mustard and its by-products, fermented by LAB against toxigenic fungi, in cereal crops such as corn. Therefore, this work contributed to filling this literary gap.

Against this background, the study aimed to develop a biopesticide, based on fermented mustard with LAB, as a solution to reduce fungal contamination and mycotoxin synthesis in corn ears. For this, the antifungal properties of aqueous extracts of yellow mustard (YM) and oriental mustard (OM) fermented by LAB were investigated against toxigenic *Fusarium* strains in vitro. Besides, the Cell-Free Supernatants (CFS) were characterised by determining the main phenolic compounds and organic acids produced. Finally, a biopreservative made from yellow mustard extract, fermented by *Lactiplantibacillus plantarum* TR71 was evaluated in corn ears contaminated with *F. verticillioides* to prevent the FB_1_ and FB_2_ production.

## 2. Results and Discussion

### 2.1. Antifungal Activity of the Fermented Mustard Extracts

Two water extracts prepared from different varieties of mustard, YM (*Sinapis alba*) and OM (*Brassica juncea*), were fermented by nine LAB and tested against toxigenic *Fusarium* strains in vitro. For this purpose, a qualitative assay on PDA plates was employed to initially screen the different CFS’ antifungal properties. The control group consisted of non-fermented YM and OM water extract. As plotted in Table 1, only the extracts fermented by *L. plantarum* TR7, *L. plantarum* TR71, *L. plantarum* TR14, and *L. plantarum* CECT 8962 evidenced antifungal properties. In particular, the YM extracts fermented by *L. plantarum* TR71 and *L. plantarum* TR14 showed inhibition halos larger than 10 mm against all *Fusarium* strains. The other LAB strains were tested (*Leuconostoc pseudomesenteroides* IRK751, *Levilactobacillus brevis* IRK82, *Levilactobacillus brevis* SMF76, *Leuconostoc pseudomesenteroides* POM, and *Liquorilactobacillus ghanensis* TR2), and the control extracts did not show antifungal effect. Comparing both mustard extracts, YM fermented extracts were more effective than OM fermented extracts since some fungal strains were resistant to the latter.

Therefore, based on these results, the mustard extracts fermented by *L. plantarum* strains were selected for further analysis, which consisted of a quantitative antifungal test to determine the Minimum Inhibitory Concentration (MIC) and Minimum Fungicidal Concentration (MFC) values against the *Fusarium* strains. The results obtained from MIC and MFC trials are presented in Table 2.

The MIC and MFC values varied, according to the LAB strain tested and the employed mustard variety (yellow or oriental) employed as fermentation substrate. The YM extract fermented by *L. plantarum* TR71 obtained the lower MIC and MFC values, ranging from 7.8–15.6 g/L and 15.6–31.3 g/L, respectively, i.e., the extract fermented by *L. plantarum* TR71 needed lower doses than other fermented extracts to inhibit fungal growth. In particular, the most susceptible fungal strains to this extract were *F. graminearum* ITEM 126, *F. graminearum* ITEM 6352, *F. graminearum* ITEM 6415, *F. verticillioides* ITEM 12043, *F. sporotrichioides* ITEM 121, *F. langsethiae* ITEM 11031, and *F. poae* ITEM 9151. Although the other YM extracts fermented by LAB exhibited an antifungal capacity, their MIC and MFC values were higher, ranging from 15.6–31.3 g/L and 31.3–62.5 g/L, respectively. Therefore, the antifungal activity seemed to be lower.

In general, the MIC and MFC values for the OM fermented extracts were higher than those for the YM fermented extracts, supporting the previous qualitative test findings (Table 1). Similarly, MIC values varied from 15.6–62.5 g/L according to the strain used, whereas for MFC values, the concentration needed to achieve a fungal inhibition ranged from 31.3–125.0 g/L. The higher resistance to the OM extract was obtained by *F. proliferatum* ITEM 16031, *F. verticillioides* ITEM 12044, *F. poae* ITEM 9131, *F. poae* ITEM 9151, and *F. poae* ITEM 9211, with MFC values ranging from 62.5 to 125.0 g/L, depending on the *L. plantarum* strain used in the fermentation procedure. Thus, this study demonstrated the in vitro antifungal activity of the mustard CFS after fermentation by LAB against *Fusarium* spp.

Although previous studies have confirmed the efficacy of YM and OM in preventing fungal development, the use of fermented mustard extract as an antifungal treatment method has not been reported in the literature. Quiles et al. [25] studied the antifungal properties of water extracts prepared from YM and OM flour and confirmed that YM water extract was effective against toxigenic fungi of the *Aspergillus*, *Penicillium*, and *Fusarium* genera in concentrations ranging from 0.24 to 7.5 g/L, whereas OM water extract was not antifungal. The YM extract prepared as a control in our study was not effective after incubation at 37 °C for 72 h. This finding agrees with our previous study since the antifungal properties of YM water extracts may decrease when the extract is stored for more than 24 h at a temperature higher than 25 °C [26]. Therefore, it seems that fermentation might yield more stable molecules and, hence, enhance the antifungal activity of YM and OM extracts. To be precise, the OM extract exhibited antifungal activity only when fermentation was applied.

Concerning antifungal effectiveness of the CFS, other authors have reported MIC and MFC values of LAB after fermentation of different food matrices. Luz et al. [27] evaluated the antimicrobial properties of lyophilized whey, fermented by LAB, against nine toxigenic strains of the *Penicillium*, *Aspergillus*, and *Fusarium* genera. The CFS evidenced antifungal properties regarding *Fusarium* strains, with MIC and MFC values ranging from 31.3–125 and 62.5–250 g/L, respectively.

Izzo et al. [28] determined the MIC and MFC concentration of fermented goat’s sweet whey using *Lactobacillus* spp. against ten toxigenic *Fusarium* strains. The author obtained MIC values ranging from 1.5 to 31.2 g/L, whereas the mean MFC values ranged from 7.8 to 250 g/L. It is essential to underline that our results corroborate that study since similar MIC and MFC values inhibited the growth of *Fusarium* spp. Our results, associated with previous studies, could confirm the possible application of CFS of YM as an antifungal agent against *Fusarium* strains.

### 2.2. Phenolic Acids and Organic Acids Profile of the CFS

This study characterized the main phenolic acids of the fermented mustard extracts that exhibited antifungal properties in vitro through liquid chromatography (UHPLC-qTOF/MS). There were 11 different phenolic acids identified in the CFS of the YM and OM fermented extracts. As expected, it was noted that the profile and concentration of phenolic acids differed according to the LAB strain and the mustard variety employed as substrate for the fermentation. The results are summarized in Table 3. In the YM extracts (Table 3a), 1,2-dihydroxybenzene, 3,4-dihydroxicinnamic acid, and benzoic acid were significantly increased (*p* < 0.05) after fermentation by *L. plantarum* strains compared to control extracts. In particular, *L. plantarum* TR71 produced the higher concentration of these compounds with a mean of 292.85, 44.95, and 220.12 ng/mL, respectively. Moreover, this strain synthesized 559.15 ng/mL of 3-Phenyllactic acid, the highest concentration among the assessed CFS, regardless of the mustard extract tested.

Regarding OM extracts (Table 3b), 3,4-dihydroxicinnamic acid, benzoic acid, and 3-phenyllactic acid were identified and quantified in higher concentration in comparison with the control extract (*p* < 0.05), the concentrations ranged from 146.01 to 217.67, 140.53 to 228.43, and 31.20 to 37.16 ng/mL, respectively. 

Comparing both mustard extracts, lower values of 1,2-dihydroxybenzene were detected in the OM extracts, whereas higher values of 3,4-dyhydroxicinnamic acid were quantified in this extract. Moreover, other cinnamic acid derivatives, characteristic of mustard seeds, such as p-coumaric acid, ferulic acid, and sinapic acid, were also identified in both extracts [29]. Although both extracts increased phenolic acid concentration after fermentation, the YM extract showed a slightly higher concentration than OM. These results suggested that the LAB fermentation could be beneficial, increasing the antifungal potential of YM extract and generating antifungal compounds in OM extract.

Only lactic acid was identified in the fermented samples regarding the organic acids. In particular, the lactic acid content in the YM fermented extracts ranged from 570.26–799.88 ng/mL, and the higher content of this organic compound was produced by *L. plantarum* TR71. Lactic acid was also detected on the OM fermented extract. However, the concentrations quantified were lower compared to YM extracts, with values ranging from 89.24–209.04 μg/mL.

The LAB antimicrobial potential is well-known and, for this reason, have been studied for food and feed application [22,23,30]. The antimicrobial properties of these microorganisms are not characteristic of one chemical compound; for instance, several metabolites, such as organic acids, phenolic acids, antimicrobial peptides, and fatty acids, can act synergistically and provide antifungal activity [31]. Among the metabolites produced by LAB, organic acids are considered the main compounds responsible for the biopreservative activity of LAB. Their antifungal properties are directly related to the decrease in pH, which inhibits the fungal cell’s metabolic activities and disrupts the cell membrane [14]. In this study, only lactic acid was detected in all fermented CFS, and the higher concentration of this metabolite was detected on the YM fermented extracts, which also evidenced the higher antifungal properties in the in vitro studies.

The identified compounds in our extracts have been described previously as antifungal substances in other CFS regarding the phenolic acids. Chen et al. [32] reported several phenolic compounds in CFS obtained through fermentation of *L. kefiri* M4 with antifungal properties against *P. expansum* such as 1,2-dihydroxybenzene, 3,4-dihydroxicinnamic acid, benzoic acid, and 3-phenyllactic acid. Among the identified phenolic compounds, 3-phenyllactic acid has been widely studied for its antifungal potential against mycotoxigenic fungi, and some authors have established a positive correlation between PLA content and the antifungal properties [33]. In this context, Cortes-Zavaleta et al. [34] screened 13 LAB for their ability to produce 3-phenyllactic acid and their antimicrobial properties against food spoilage moulds, such as *Botrytis cinerea*, *Penicillium expansum*, and *Aspergillus flavus*. They correlated the antifungal properties of the LAB regarding the 3-phenyllactic acid synthesized. However, the authors agree that further investigation should be done since the antifungal properties are not exclusively related to this phenolic compound. Therefore, the higher content of 3-phenyllactic acid synthesized in YM extracts fermented by *L. plantarum* TR71 could be related to the higher antifungal properties. Due to the higher in vitro *antifungal activity*, this extract was proposed as an antimycotoxigenic agent in corn ears contaminated with *F. verticillioides*.

### 2.3. Application of the CFS on Corn Ears as an Antimycotoxigenic Agent

**Figure 1:**
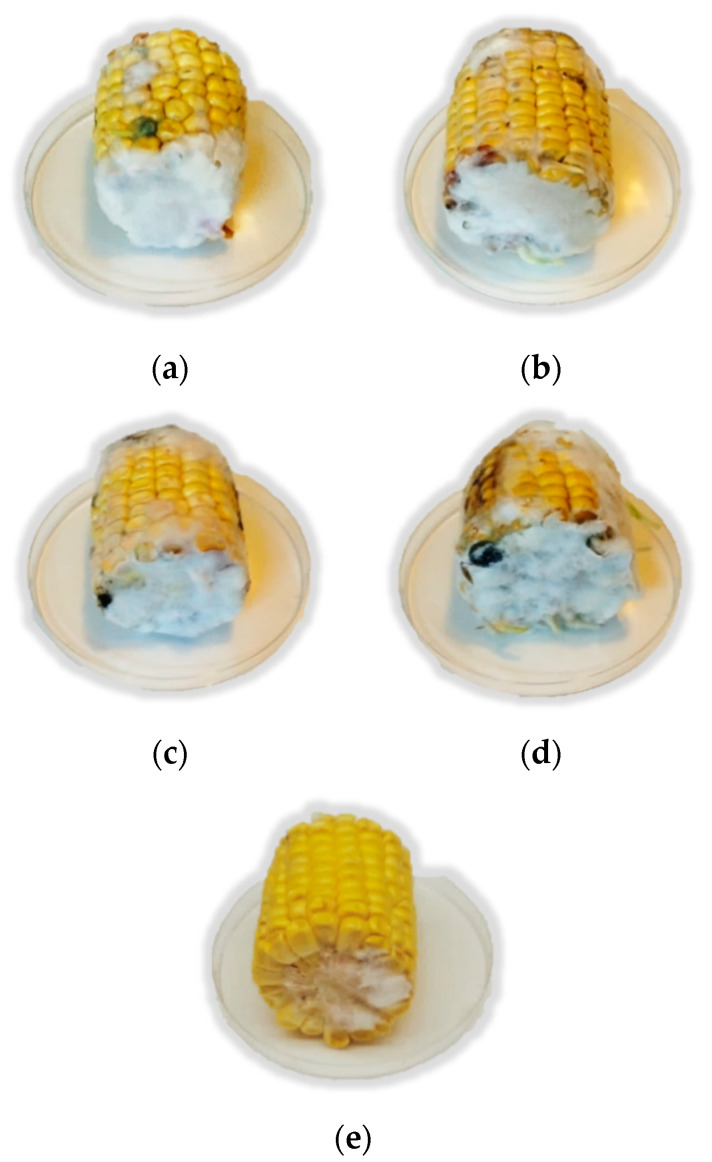
Corn ears contaminated with *F. verticillioides* ITEM 12,052 after 14 days of storage. Treatments applied were the following: (**a**) control; (**b**) non-fermented yellow mustard extract; (**c**) fermented yellow mustard extract with *L. plantarum* TR71; (**d**) non-fermented yellow mustard extract lyophilized and prepared at 250 g/L in water; (**e**) fermented yellow mustard extract with *L. plantarum* TR71, lyophilized and prepared at 250 g/L in water.

**Figure 2:**
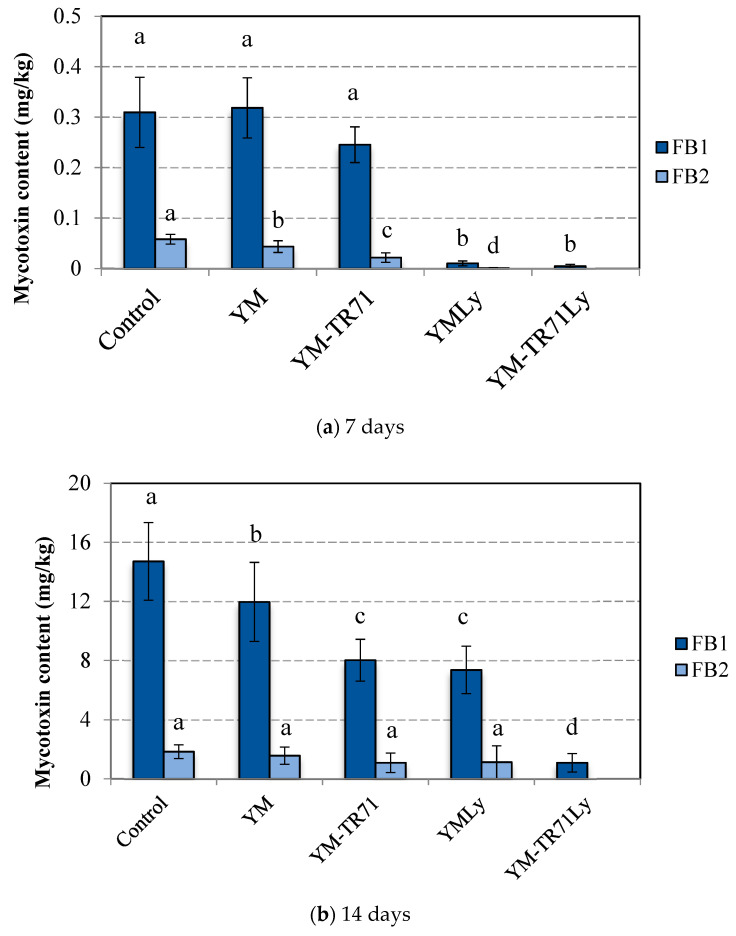
Fumonisin B_1_ (FB_1_) and Fumonisin B_2_ (FB_2_) determination in corn ears contaminated with *F. verticillioides* ITEM 12,052 and treated with cell-free supernatant. Treatments applied were the following: YM: non-fermented yellow mustard extract; YM-TR71: fermented yellow mustard extract with *L. plantarum* TR71; YMLy: non-fermented yellow mustard lyophilized and prepared at 250 g/L; YM-TR71Ly: fermented yellow mustard with *L. plantarum* TR71 lyophilized and prepared at 250 g/L. Mycotoxin was determined on 7th (**a**) and 14th day (**b**). The different letter means statistical differences in the mycotoxin content among the treatments applied (*p <* 0.05).

The YM fermented extract with *L. plantarum* TR71 was selected and applied as a bio-preservative against *F. verticillioides* (FB_1_ and FB_2_ producer) in corn ears. For this purpose, the fermented YM extract with *L. plantarum* TR71 was applied directly through the spray technique on the corn ears, or after lyophilization and preparation, at 350 g/L in sterile water. In addition, the YM extract was also tested on the corn ears without fermentation (through direct spray or lyophilization and preparation at 350 g/L). The control group was prepared with non-treated corn ears inoculated with the fungal agent. Then, the corn ears were stored at 25 °C for 14 days (Figure 1), determining the mycotoxin content at times 0, 7, and 14 days (Figure 2) through the UHPLC Q-TOF/MS technique.

At the initial time (0 d), the samples did not show mycotoxins. After 7 days post-inoculation (Figure 2a), the control contained 0.30 mg/kg of FB_1_ and 0.05 mg/kg of FB_2_. Furthermore, only the administration of lyophilized extracts (fermented or unfermented) demonstrated a decrease in FB_1_ levels, as compared to the control treatment (*p* < 0.05). Additionally, the lyophilized extract fermented by TR71 was the only treatment that did not evidence FB_2_ production after 7 days of incubation.

After 14 days (Figure 2b), the FB_1_ synthesized by *F. verticillioides* increased in all the treatments tested. The FB_1_ content (without treatment) was raised to 14.71 mg/kg in the control group. In contrast, we noticed that the direct application of the YM extract fermented by TR71 reduced the FB_1_ production (8.02 mg/kg) compared to the control (49.5% of reduction), and, similarly, the unfermented lyophilised YM significantly reduced the FB_1_ concentration regarding the control group. Nevertheless, it is worth noting that the higher decrease in FB_1_ was achieved by applying the lyophilised YM extract fermented by TR71. Remarkably, the average content obtained after application of this treatment and incubation for 14 days was 1.09 mg/kg, which, compared to the control, reduced the incidence of this mycotoxin in corn ears by 92.6%.

The antimycotoxigenic effect of the fermented YM extract could be explained because the application of the CFS reduced the fungal growth (Figure 1), and, in consequence, the secondary metabolism responsible for the mycotoxin synthesis could be retarded [35]. Regarding FB_2_, non-statistically differences (*p* < 0.05) were evidenced between the spray with mustard and the control group, except for the lyophilised YM fermented by TR71. This treatment completely inhibited FB_2_ synthesis by *F. verticillioides* on corn ears. 

The treatments applied could not wholly reduce the production of FB_1_ on corn ears. However, it is essential to underline that, after 14 days of incubation, the mycotoxin content, in corn ears treated with lyophilized *L. plantarum* TR71, was below 4 mg/kg, which means below the maximum levels specified in the European legislation for the sum of FB_1_ and FB_2_ in unprocessed corn [36]. Thus, YM fermented with TR71 was proved to be an antimycotoxogenic solution for corn ears, and we suggest its application during pre-harvest to increase food safety.

The present trend toward minimizing the use of agrochemicals in food has prompted researchers to investigate alternative strategies for lowering the occurrence of toxigenic fungal agents. Several authors have confirmed the promising employment of LAB to avoid mycotoxins production in food and feed. In this context, Nazareth et al. [37] evaluated the application of the CFS prepared from fermented MRS broth with *L. plantarum* CECT 749 against *F. verticillioides* and *Aspergillus flavus* in corn ears and corn kernels, respectively. Although they did not completely reduce the incidence of FB_1_, the content of this mycotoxin on corn ears decreased 90.6% after 7 days compared to the control. In corn kernels, the effect of applying the CFS reduced the incidence of aflatoxin B_1_ by 99.7 and 97.5% after 25 and 40 days, respectively.

Dopazo et al. [38] isolated and studied the use of LAB on red grapes as bio-preservative agents against *A. flavus*, *A. niger*, and *Botrytis cinerea*. They found that the use of *L. fallax* UTA 6 CFS was effective against *A. flavus* and *B. cinerea*, reducing the fungal population on red grapes by 0.4 and 0.6 log spores per gram. Additionally, they investigated the efficacy of CFS treatment in reducing mycotoxin occurrence on red grapes, and the authors observed that aflatoxin B_1_ and fumonisins (B_2_, B_3_, and B_4_) were reduced in percentages ranging from 28 to 100%.

Ben Taheur et al. [39] applied the CFS obtained by LAB in almonds against *A. flavus* and *A. carbonarius*. The use of the CFS of *L. kefiri* FR7 reduced the incidence of aflatoxin B_1_ and aflatoxin B_2_, synthesized by *A. flavus* in 85.27% and 83.94%, respectively. Moreover, a similar effect was observed when the inoculant agent was *A. carbonarius*, since the Ocratoxin A content was reduced 25% compared to the control.

## 3. Conclusions

In this study, the fermented mustard extracts by LAB were proposed as a natural biopreservative solution in corn ears. The in vitro evaluation of the antifungal properties showed that the YM extracts fermented by *L. plantarum* strains presented the highest antifungal effect against *Fusarium* spp.

Although 11 different phenolic acids were identified, the characterization of the CFS highlighted that lactic acid and 3-phenyllactic acid were the most abundant antifungal metabolites in the YM extract fermented by *L. plantarum* TR71. Therefore, due to the higher in vitro antifungal activity, as well as lactic and phenolic acid production, this extract was applied on corn ears contaminated with *F. verticillioides* to reduce FB_1_ and FB_2_ production.

In conclusion, the fermented YM extract effectively reduced more than 90% of FB_1_ and FB_2_ content after 14 days of incubation. Since consumers are demanding a reduction in pesticides to preserve crops, the proposed application of YM fermented extracts by *L. plantarum* TR71 is a sustainable solution that reduces the incidence of mycotoxin contamination and, hence, increases the food safety of corn ears. Finally, we recommend its application against different fungal contaminants in the field to evaluate its capacity to avoid the production of different mycotoxins.

Further studies should be developed using this biopreservation associated with different barrier technologies such as temperature control, water activity, application of other natural compounds, or modified atmosphere packaging. Using one or several barriers would probably increase crop quality, reducing the Fumonisin production to undetectable levels. 

## 4. Materials and Methods

### 4.1. Chemicals

The FB_1_ standard solution (purity > 99%) was obtained from Sigma–Aldrich (St. Louis, MO, USA). The phenolic standards 1,2-dihydroxybenzene, 3,4-dihydroxicinnamic acid, benzoic acid, 3-phenyllactic acid, hydroxycinnamic acid, p-coumaric acid, protocatechuic, sinapic acid, vanillin, syringic acid, and ferulic acid were purchased from Sigma–Aldrich (St. Louis, MO, USA). Lactic acid was obtained from Sigma–Aldrich (St. Louis, MO, USA).

Acetonitrile (ACN) (LC-MS/MS grade), ethyl acetate (EA), formic acid (FA), and methanol (HPLC-MS/MS grade) were obtained from VWR Chemicals (Randor, PA, USA). The deionised water used in chromatography analysis (<18 MΩ cm resistivity) was obtained from a Milli-Q purification system (Millipore, Bedford, MA, USA). The salts, magnesium sulphate (MgSO_4_) and sodium chloride (NaCl), were provided from Sigma–Aldrich (St. Louis, MO, USA).

The Potato Dextrose Agar (PDA), Potato Dextrose Broth (PDB), and Buffered peptone water (BPW) were purchased from Liofilchem Bacteriology Products (Roseto, Italy). De man Rogosa Sharpe (MRS) Broth was obtained from Oxoid (Hampshire, UK).

The Yellow Mustard Flour (YM) (code #106) and Oriental Mustard Flour (OM) (code #107) were provided by G.S. Dunn Dry Mustard Millers (Hamilton, ON, Canada).

### 4.2. Microorganisms and Culture Conditions

The fungal strains *Fusarium graminearum* ITEM 126, *F. graminearum* ITEM 6352, *F. graminearum* ITEM 6415, *F. proliferatum* ITEM 12072, *F. proliferatum* ITEM 12103, *F. proliferatum* ITEM 16031, *F. verticillioides* ITEM 12052, *F. verticillioides* ITEM 12043, *F. verticillioides* ITEM 12044, *F. sporotrichioides* ITEM 12168, *F. langsethiae* ITEM 11031, *F. poae* ITEM 9131, *F. poae* ITEM 9151, and *F. poae* ITEM 9211 were obtained from the Institute of Sciences of Food Production (ISPA-CNR, Bari, Italy). The fungi were preserved in sterile PDB 25% glycerol at −80 °C. Prior to their use, the strains were transferred into PDA plates and incubated for 7 d at 25 °C. The spores collected from these plates were used in the study.

The LAB strain *Leuconostoc pseudomesenteroides* IRK751, *Levilactobacillus brevis* IRK82, *Levilactobacillus brevis* SMF76, *Leuconostoc pseudomesenteroides* POM, *Lactiplantibacillus plantarum* TR7, *Lactiplantibacillus plantarum* TR71, *Lactiplantibacillus plantarum* TR14, and *Liquorilactobacillus ghanensis* TR2 were isolated from tomatoes and sourdough and identified through the 16S rNA analysis sequence by Luz et al. [40]. The strain *Lactiplantibacillus plantarum* CECT 8962 was obtained from the Spanish Culture Type Collection CECT (Valencia, Spain). The LAB strains were recovered from MRS 25% glycerol stored at −80 °C and inoculated in fresh MRS broth for 72 h at 37 °C.

### 4.3. Fermentation Conditions and Preparation of CFS

The mustard extracts used for fermentation were prepared, according to Quiles et al. [25], with minor modifications. Firstly, 10 g of YM or OM were mixed with 250 mL of distilled water and homogenised using an Ultraturrax T18 basic mixer (Ika, Staufen, Germany) and then centrifuged at 4000× *g* for 15 min at 4 °C. The supernatant obtained was used for bacteria fermentation, as follows. Next, 1 mL of each LAB (10^7^ CFU/mL) described by Section 4.2 and growth in MRS for 12 h (to achieve the exponential phase growth) was added to 9 mL of the YM or OM water extract (proportion 1:10 *v*/*v*), homogenised, and incubated for 72 h at 37 °C. Control extracts were prepared without adding LAB. Then, the fermented extracts were centrifuged at 3200 × *g* for 10 min to obtain the CFS. Part of the extract was lyophilised (FreeZone 2.5 L, Labconco, Kansas City, MO, USA) and stored at −30 °C to characterise the antifungal properties. The liquid CFS was used to determine the organic acids and phenolic acids profile.

### 4.4. Qualitative Antifungal Test on PDA Plates

The lyophilised CFS were prepared at a final concentration of 100 g/L with sterile water and tested on PDA plates against the *Fusarium* fungal strains described by Section 4.2. The fungal spores were collected with a cotton swab, soaked with 0.1% buffered peptone water 0.2% TWEEN^®^ and cultivated on PDA plates. Then, wells of 10 mm diameter were prepared in the agar, and 100 µL of the CFS was placed. The plates were incubated at 25 °C for 48 h to observe fungal inhibition. The inhibition on the fungal growth was considered positive (+) when the inhibition zone was more extensive than 10 mm in diameter. The control was realised by testing the extracts of YM and OM without fermentation.

### 4.5. Determination of the MIC and MFC Values of the CFS

The Minimum Inhibitory Concentration (MIC) and the Minimum Fungicidal Concentration (MFC) of the mustard-fermented CFS were established, according to the CLSI document M38-A2 [41], with modifications. The lyophilized extracts were mixed with PDB, and 100 μL were assayed in 96-well microplates at concentrations ranging from 7.8 to 200 g/L. In addition, two controls were prepared on each microplate. The first one constituted the negative control, which contained only 200 μL of PDB. The second control, the control of the microorganism, was prepared, adding to the plate the fungal strains described by Section 4.2 without the antifungal agent. The fungal spores were collected from PDA plates and, with a cotton swab, counted with a Neubauer chamber and adjusted to 5 × 10^4^ spores/mL in PDB. Next, 100 μL of the fungal spores were added to the wells containing the antifungal agent, so the final volume was 200 μL/well. The plates were incubated 72 h at 25 °C, and the MIC was established as the smallest concentration of the antifungal agent that inhibited the fungal growth compared to the control of the microorganism.

After determination of the MIC, 10 μL of the higher doses of the MIC were subcultured on PDA plates and incubated 48 h at 25 °C. Finally, the MFC value was considered the lowest concentration in which fungal growth was not detected on the PDA plate.

### 4.6. Organic Acids and Phenolic Acids Determination in the CFS

For the determination of the organic acids, the mustard CFS was diluted 1:20 (*v*/*v*) in Milli-Q water and then filtered with a 0.22 µm syringe filter. The samples were injected into an Agilent 1100 Series HPLC System (Palo Alto, CA, USA), equipped with a diode array detector and a quaternary pump. The separation was realised with a Rezez ROA-Organic Acid (140 × 7.8 mm) reverse phase column (Phenomenex, Torrance, CA, USA). The isocratic mobile phase used was water 0.1% FA (*v*/*v*) with a flow rate of 0.6 mL/min. The chromatogram was monitored at 210 nm [42]. The results were expressed in ng/mL Three replicates (*n* = 3) of each extract were analysed and the experiment was conducted three times.

The extraction of the phenolic acids from the mustard CFS was realised following the methodology of Brosnan et al. [43]. There was 10 mL of the CFS incorporated in Falcon tubes together with 10 mL of EA 1% FA, 4 g of MgSO_4_, and 1 g of NaCl. Then, the tubes were mixed by vortex for 1 min and kept on ice for 5 min. To separate the ethylic phase, the tubes were centrifuged. Afterwards, the ethylic phase was transferred to a new Falcon tube, containing 150 mg of C18 and 900 mg of MgSO_4_, and the mixture was vortexed again for 1 min. Then, the samples were centrifuged, and the supernatant recovered was dried under N_2_ flow. The samples were reconstituted with 1 mL of Milli-Q water: ACN (50:50 *v*/*v*) and filtered with a 0.22 µm syringe filter before the injection on the LC system.

The analysis of the phenolic acids was realised using a 6450 Agilent Ultra High-Definition Accurate Mass QTOF-MS, equipped with an Agilent Dual Jet Stream Electrospray Ionization. The column employed for chromatographic separation was a Gemini C18 (50 mm × 2 mm, 100 Å, 3 μm particle size) (Phenomenex, Torrance, CA, USA), and the mobile phases used were Milli-Q water (phase A) and ACN (phase B), both acidified with FA 0.1%. The gradient elution was programmed as follows: 0 min, 5% B; 30 min, 95% B; 35 min, 5% B. The equilibration of the column was set at 3 min before the following analysis. There were 20 μL of the samples injected, and the flow rate was 0.3 mL/min.

The mass spectrometry analyses were conducted in negative ionisation mode with the following conditions: drying gas (N_2_), 8.0 L/min; nebuliser pressure, 30 psig; gas drying temperature, 350 °C; capillary voltage, 3.5 kV; fragmentation voltage, 175 V; scan range, 20–380 m/z. Collision energies for MS/MS experiments were 10, 20, and 40 eV. The integration and data elaboration was realised with MassHunter Qualitative Analysis Software B.08.00 [44]. Results were expressed in ng/mL. Three replicates (*n* = 3) of each extract were analysed, and the experiment was conducted three times.

### 4.7. Application of the CFS in Corn Ears

The antimycotoxigenic activity of the YM CFS fermented by *L. plantarum* TR71 was studied on corn ears contaminated with *F. verticillioides* CECT 2982 (FB_1_ and FB_2_ producer). Samples of corn ears (*Zea mays* L. var. *rugosa*) (70 g), purchased from a local supermarket, were placed in 1L glass jars. Then, corn ears were treated by spraying 2 mL of the fermented YM extract by *L. plantarum* TR71 or 2 mL of the YM extract lyophilised, preparing the solutions at a concentration of 350 g/L in sterile water. Moreover, the non-fermented YM extract was prepared and applied in the same conditions: 2 mL of YM extract; 2 mL of the YM extract, lyophilised and prepared at 350 g/L. A control treatment was designed by spraying 2 mL of sterile water on the corn ears. Then, 1 mL of *F. verticillioides* CECT 2982, prepared at 10^3^ spores/mL in 0.1% buffered peptone water, was sprayed on the corn earns and was let dry for 1 h. Afterwards, the jars were closed and stored at 25 °C for 14 days. Nine replicates (*n* = 9) of each treatment were prepared, and the study was conducted three times.

### 4.8. Extraction and Determination of Mycotoxins by Q-TOF

The extraction of FB_1_ and FB_2_ was realised using the methodology described by Nazareth et al. [37] with modifications. Before the extraction, the lyophilised corn portions were finely grounded with an Oester Classic grinder (Madrid, Spain). Then, 5 g were mixed with 25 mL of methanol, and the extraction was performed in an Ultraturrax at 12,000 rpm for 5 min. Next, the mixture obtained was centrifuged, and then, the supernatant was recovered, filtered through a 0.22 µm syringe filter and injected into a UHPLC (1290 Infinity LC, Agilent Technologies) coupled to a Q-TOF (Agilent 6540 LC/QTOF) Mass Spectrometer. 

The chromatographic separation of the mycotoxins was realised in an Agilent Zorbax RRHD SB-C18 (2.1 × 50 mm, 1.8 μm) column. Mobile phases employed were the following: Milli-Q water 0.1% FA (Phase A); ACN 0.1% FA (Phase B). The gradient used was configured as: 0 min, 2% B; 22 min, 95% B; 25 min, 5% B. Then, the column was equilibrated 3 min before the next injection. Flow rate was established at 0.4 mL/min, and the injection volume was 5 μL. For q-TOF analysis, an Agilent Dual Jet Stream electrospray ionisation (ESI) was operating in positive ionisation mode. Conditions of ESI were configured as follows: gas temperature: 325 °C; gas flow: 10 L/min; nebuliser pressure: 40 psig; sheath gas temperature: 295 °C; sheath gas flow: 12 L/min; capillary voltage: 4000 V; nozzle voltage: 500 V; skimmer: 70 V; scan range: 100–1500 Da; collision energy: 10, 20, 40 eV. For quantification, fumonisin calibration curves were prepared, with concentration ranging from 0.01 to 10 mg/L. The integration and data elaboration were realised using MassHunter Qualitative Analysis Software B.08.00.

### 4.9. Statistical Analysis

For statistical analysis, GraphPad Prism version 3.0 software (San Diego, CA, USA) was used. The differences between groups (*p* < 0.05) were analysed by One-Way ANOVA test followed by the post-hoc Tukey test for multiple comparisons. Results were expressed as mean ± SD.

## Figures and Tables

**Table 1:** Antifungal activity of the Cell-Free Supernatant (CFS) at 100 g/L against toxigenic *Fusarium* strains. Two mustard varieties were employed as fermentation substrates: Yellow Mustard (YM) and Oriental Mustard (OM). Antifungal activity was considered positive (+) when the inhibition halo measurement was more extensive than 10 mm.

Fungal Strain	Control		IRK751		IRK82		SMF76		POM		TR7		TR71		TR14		TR2		CECT 8962	
	YM	OM	YM	OM	YM	OM	YM	OM	YM	OM	YM	OM	YM	OM	YM	OM	YM	OM	YM	OM
*F. graminearum* ITEM 126	−	−	−	−	−	−	−	−	−	−	+	−	+	−	+	+	−	−	+	−
*F. graminearum* ITEM 6352	−	−	−	−	−	−	−	−	−	−	−	−	+	−	+	+	−	−	+	+
*F. graminearum* ITEM 6415	−	−	−	−	−	−	−	−	−	−	+	−	+	−	+	−	−	−	+	−
*F. proliferatum* ITEM 12072	−	−	−	−	−	−	−	−	−	−	+	+	+	+	+	+	−	−	+	+
*F. proliferatum* ITEM 12103	−	−	−	−	−	−	−	−	−	−	−	−	+	−	+	−	−	−	−	−
*F. proliferatum* ITEM 16031	−	−	−	−	−	−	−	−	−	−	+	+	+	+	+	+	−	−	+	+
*F. verticillioides* ITEM 12052	−	−	−	−	−	−	−	−	−	−	+	+	+	+	+	+	−	−	+	+
*F. verticillioides* ITEM 12043	−	−	−	−	−	−	−	−	−	−	+	+	+	+	+	+	−	−	+	+
*F. verticillioides* ITEM 12044	−	−	−	−	−	−	−	−	−	−	+	+	+	+	+	+	−	−	+	+
*F. sporotrichioides* ITEM 121	−	−	−	−	−	−	−	−	−	−	+	−	+	−	+	+	−	−	−	−
*F. langsethiae* ITEM 11031	−	−	−	−	−	−	−	−	−	−	+	−	+	+	+	+	−	−	+	+
*F. poae* ITEM 9131	−	−	−	−	−	−	−	−	−	−	−	−	+	−	+	−	−	−	+	−
*F. poae* ITEM 9151	−	−	−	−	−	−	−	−	−	−	+	−	+	−	+	−	−	−	+	−
*F. poae* ITEM 9211	−	−	−	−	−	−	−	−	−	−	+	−	+	+	+	+	−	−	+	+

Leuconostoc pseudomesenteroides IRK751; Levilactobacillus brevis IRK82; Levilactobacillus brevis SMF76; Leuconostoc pseudomesenteroides POM; Lactiplantibacillus plantarum TR7; Lactiplantibacillus plantarum TR71, Lactiplantibacillus plantarum TR14; Liquorilactobacillus ghanensis TR2; Lactiplantibacillus plantarum CECT 8962.

**Table 2:** Minimum Inhibitory Concentration (MIC) and Minimum Fungicidal Concentration (MFC) values determined in vitro against toxigenic *Fusarium* strains of (**a**) Yellow Mustard fermented cell-free supernatant; (**b**) Oriental Mustard fermented cell-free supernatant. Results were expressed as g/L.

**(a)**								
**Fungal Strain**	**TR7**		**TR71**		**TR14**		**CECT 8962**	
	**MIC**	**MFC**	**MIC**	**MFC**	**MIC**	**MFC**	**MIC**	**MFC**
*F. graminearum* ITEM 126	15.6	31.3	7.8	15.6	7.8	15.6	31.3	62.5
*F. graminearum* ITEM 6352	15.6	31.3	7.8	15.6	15.6	31.3	15.6	31.3
*F. gramienarum* ITEM 6415	7.8	15.6	7.8	15.6	7.8	15.6	7.8	15.6
*F. proliferatum* ITEM 12072	31.3	62.5	7.8	15.6	15.6	31.3	15.6	31.3
*F. proliferatum* ITEM 12103	15.6	31.3	15.6	31.3	15.6	31.3	15.6	31.3
*F. proliferatum* ITEM 16031	15.6	62.5	15.6	31.3	31.3	62.5	15.6	31.3
*F. verticillioides* ITEM 12052	15.6	31.3	15.6	31.3	31.3	62.5	15.6	31.3
*F. verticillioides* ITEM 12043	7.8	15.6	7.8	15.6	15.6	31.3	15.6	31.3
*F. verticillioides* ITEM 12044	15.6	31.3	15.6	31.3	31.3	62.5	15.6	31.3
*F. sporotrichioides* ITEM 121	31.3	62.5	7.8	15.6	7.8	15.6	15.6	31.3
*F. langsethiae* ITEM 11031	15.6	31.3	7.8	15.6	7.8	15.6	7.8	15.6
*F. poae* ITEM 9131	15.6	31.3	15.6	31.3	15.6	31.3	15.6	31.3
*F. poae* ITEM 9151	7.8	15.6	7.8	15.6	15.6	31.3	31.3	62.5
*F. poae* ITEM 9211	15.6	31.3	15.6	31.3	15.6	31.3	31.3	62.5
**(b)**								
**Fungal Strain**	**TR7**		**TR71**		**TR14**		**CECT 8962**	
	**MIC**	**MFC**	**MIC**	**MFC**	**MIC**	**MFC**	**MIC**	**MFC**
*F. graminearum* ITEM 126	31.3	62.5	31.3	62.5	15.6	62.5	31.3	62.5
*F. graminearum* ITEM 6352	31.3	62.5	31.3	62.5	31.3	62.5	15.6	31.3
*F. gramienarum* ITEM 6415	15.6	31.3	15.6	31.3	15.6	62.5	15.6	31.3
*F. proliferatum* ITEM 12072	31.3	62.5	31.3	62.5	31.3	62.5	31.3	62.5
*F. proliferatum* ITEM 12103	31.3	62.5	31.3	62.5	31.3	62.5	31.3	62.5
*F. proliferatum* ITEM 16031	62.5	125.0	31.3	62.5	31.3	62.5	62.5	125.0
*F. verticillioides* ITEM 12052	31.3	62.5	31.3	62.5	31.3	62.5	31.3	62.5
*F. verticillioides* ITEM 12043	31.3	62.5	31.3	62.5	31.3	62.5	31.3	62.5
*F. verticillioides* ITEM 12044	31.3	62.5	31.3	62.5	62.5	125.0	31.3	62.5
*F. sporotrichioides* ITEM 121	31.3	62.5	31.3	62.5	31.3	62.5	31.3	62.5
*F. langsethiae* ITEM 11031	15.6	31.3	15.6	31.3	7.8	15.6	15.6	31.3
*F. poae* ITEM 9131	31.3	125.0	62.5	125.0	62.5	125.0	31.3	62.5
*F. poae* ITEM 9151	31.3	62.5	31.3	62.5	31.3	62.5	62.5	125.0
*F. poae* ITEM 9211	31.3	62.5	31.3	62.5	62.5	125.0	62.5	125.0

Leuconostoc pseudomesenteroides IRK751; Levilactobacillus brevis IRK82; Levilactobacillus brevis SMF76; Leuconostoc pseudomesenteroides POM; Lactiplantibacillus plantarum TR7; Lactiplantibacillus plantarum TR71, Lactiplantibacillus plantarum TR14; Liquorilactobacillus ghanensis TR2; Lactiplantibacillus plantarum CECT 8962.

**Table 3:** Phenolic and organic acids identify in the cell-free supernatant of the yellow mustard extract (**a**) and the oriental mustard extract (**b**). Results are expressed in ng/mL.

**(a) Yellow mustard extract**					
**Phenolic Acid**	**Control**	**TR7**	**TR71**	**TR14**	**CECT 8962**
1,2-Dihydroxybenzene	62.88 ± 10.86 ^a^	259.53 ± 11.12 ^b^	292.85 ± 62.97 ^b^	194.99 ± 64.37 ^c^	250.17 ± 5.76 ^b^
3,4-Dihydroxicinnamic acid	11.45 ± 3.67 ^a^	32.73 ± 4.13 ^b^	44.95 ± 9.76 ^c^	26.01 ± 5.26 ^b^	28.76 ± 8.24 ^b^
Benzoic acid	14.74 ± 2.36 ^a^	134.42 ± 5.09 ^b^	220.12 ± 27.12 ^c^	128.19 ± 11.15 ^b^	127.29 ± 10.51 ^b^
3-Phenyllactic acid	13.67 ± 1.68 ^a^	45.66 ± 6.87 ^ab^	559.15 ± 78.51 ^c^	57.48 ± 12.07^b^	45.62 ± 8.16 ^ab^
Hydroxycinnamic acid	6.84 ± 0.93	n.d	n.d	n.d	n.d
P-Coumaric acid	16.13 ± 6.07 ^a^	42.66 ± 5.37 ^b^	76.07 ± 15.81 ^c^	65.25 ± 7.68 ^cd^	62.13 ± 11.97 ^d^
Protocatechuic	31.13 ± 7.60 ^a^	158.68 ± 12.96 ^b^	17.25 ± 2.11 ^c^	8.96 ± 3.69 ^c^	39.08 ± 17.86 ^a^
Sinapic acid	61.72 ± 4.86 ^a^	8.29 ± 2.39^b^	16.79 ± 0.12 ^c^	28.40 ± 7.78 ^d^	40.50 ± 8.51 ^e^
Vanillin	4.17 ± 1.56 ^a^	n.d	30.28 ± 7.92 ^b^	17.75 ± 6.06 ^c^	20.71 ± 7.62 ^c^
Syringic acid	4.30 ± 1.34	n.d	n.d	n.d	n.d
Ferulic acid	11.69 ± 4.69	n.d	n.d	n.d	n.d
**Organic acid**					
Lactic acid	n.d	728.00 ± 20.97 ^a^	799.88 ± 27.08 ^b^	591.56 ± 25.50 ^c^	570.26 ± 29.64 ^c^
**(b) Oriental mustard extract**					
**Phenolic Acid**	**Control**	**TR7**	**TR71**	**TR14**	**CECT 8962**
1,2-Dihydroxybenzene.	7.23 ± 2.64 ^a^	n.d	39.27 ± 6.70 ^b^	34.62 ± 5.39 ^b^	n.d
3,4-Dihydroxicinnamic acid	2.83 ± 0.98 ^a^	146.01 ± 16.30 ^b^	217.67 ± 35.00 ^c^	190.56 ± 52.87 ^cd^	157.06 ± 18.40 ^bd^
Benzoic acid	76.51 ± 8.85 ^a^	140.53 ± 30.09 ^b^	159.10 ± 8.99 ^b^	228.43 ± 16.90 ^c^	143.08 ± 36.13 ^b^
3-Phenyllactic acid	6.65 ± 2.87 ^a^	34.68 ± 3.37 ^bc^	37.16 ± 4.57 ^bc^	42.43 ± 14.18 ^b^	31.20 ± 7.27 ^c^
Hydroxycinnamic acid	10.14 ± 4.04	n.d	n.d	n.d	n.d
P-Coumaric acid	58.09 ± 15.02 ^ab^	68.29 ± 20.46 ^ac^	76.50 ± 8.62 ^d^	30.70 ± 13.59 ^ce^	42.44 ± 10.27 ^be^
Protocatechuic	20.30 ± 1.33 ^a^	44.24 ± 13.22 ^b^	32.23 ± 9.96 ^c^	31.33 ± 2.52 ^c^	33.39 ± 4.15 ^c^
Sinapic acid	62.37 ± 27.75 ^a^	25.09 ± 2.96 ^b^	22.59 ± 6.09 ^b^	48.21 ± 11.49 ^a^	19.18 ± 4.12 ^b^
Vanillin	9.47 ± 1.24 ^a^	18.64 ± 2.57 ^a^	60.14 ± 19.03 ^b^	58.86 ± 10.49 ^b^	59.47 ± 11.65 ^b^
Syringic acid	6.81 ± 1.45	n.d	n.d	n.d	n.d
Ferulic acid	19.74 ± 7.55	n.d	n.d	n.d	n.d
**Organic acid**					
Lactic acid	n.d	209.04 ± 66.16 ^a^	203.39 ± 6.53 ^a^	89.24 ± 20.20 ^b^	174.55 ± 7.26 ^a^

n.d = no detected; Different letters represent statistical differences between the treatments (*p* ≤ 0.05) (*n* = 9). *Lactiplantibacillus plantarum* TR7; *Lactiplantibacillus plantarum* TR71; *Lactiplantibacillus plantarum* TR14; *Lactiplantibacillus plantarum* CECT 8962.

## Data Availability

The data used to support the findings are included within the article.
